# Determinants of Inflation Expectations in the Euro Area

**DOI:** 10.1007/s10272-022-1037-6

**Published:** 2022-04-08

**Authors:** Richhild Moessner

**Affiliations:** Monetary and Economic Department, Bank for International Settlements, Centralbahnplatz 2, CH - 4002 Basel, Switzerland

**Keywords:** E52, E58

## Abstract

Euro area inflation has been rising strongly in the wake of the COVID-19 pandemic, giving rise to concerns that there could be second-round effects, with higher inflation leading to higher inflation expectations, which in turn lead to higher inflation. This could result in more persistent rises in inflation.

Inflation expectations matter for inflation in economic models commonly used for monetary policy analysis (Clarida et al., 1999; Smets, 2003; Woodford, 2003; Levin and Moessner, 2005; Moessner, 2021). The recent debate on monetary policy frameworks, including average inflation targeting, focused on the role of inflation expectations, e.g. in the monetary policy strategy reviews of the US Federal Reserve and the European Central Bank (ECB) (Powell and Wessel, 2020; Eurosystem work stream on inflation expectations, 2021). The decisions by the Federal Reserve to adopt an average inflation targeting framework in 2020, and by the ECB to change to a symmetric inflation target of 2% in 2021, were partly based on the role of inflation expectations. Policymakers also considered inflation expectations to be important for economic outcomes and monetary policy decisions in the past, despite difficulties in identifying their determinants: Former chair of the Federal Reserve Alan Greenspan noted, "I am not saying what that [inflation expectations] is a function of. We know it's a very difficult issue, but that is the key variable. It's important, but just because we can't make a judgment as to what these driving forces are in an econometric sense doesn't mean that it's not real" (Federal Open Market Committee, 1994; Coibion et al., 2018).

The literature on inflation expectation formation mechanisms has been reviewed in Coibion et al. (2018), who argue that survey-based expectations should be included more systematically in macroeconomic studies. Shiller (1978) noted the importance of studying the determination of inflation expectations. A recent review of the literature on the determinants of inflation expectations in advanced and emerging economies and new evidence can be found in Kose et al. (2020). They conclude that empirical studies on inflation expectations have mostly focused on advanced economies, and on testing the implications of the theoretical literature and evaluating the degree of anchoring of expectations.[1]

The Eurosystem work stream on inflation expectations (Eurosystem work stream on inflation expectations, 2021) has recently studied the determinants of inflation expectations in the euro area within the ECB's monetary policy strategy review, but without considering the effects of exchange rate changes. They find that short-term euro area inflation expectations have been affected by oil prices, monetary policy shocks and central bank inflation projections. Galati et al. (2018) also find that short-term inflation expectations in the euro area have been affected by oil prices. Household inflation expectations in the United States and Japan responded to changes in food and oil prices (Ueda, 2010). Gerlach et al. (2011) find that short-term inflation expectations in major advanced and emerging economies have been affected by food, energy and core consumer price inflation. Patra and Ray (2010) find that lagged inflation, movements in food and fuel prices and the output gap are the main determinants of short-term inflation expectations in India. They note that the scarce literature on the determinants of inflation expectations in emerging economies has mainly focused on target credibility or the role of fiscal expectations (Minella et al., 2003; Celasun et al., 2004).

Our paper contributes to a better understanding of the determinants of short-term inflation expectations in the euro area. As possible determinants of inflation expectations, we consider food and energy prices, both country-specific measures in the form of components of the consumer price index (CPI), and global food commodity prices and oil prices. We also consider the effects of changes in exchange rates and global freight prices. Moreover, we include the output gap as an explanatory variable.

* The views in this paper are those of the author and do not necessarily reflect those of the Bank for International Settlements. I am grateful to Emese Kuruc for excellent help with the data.

**Richhild Moessner**, Bank for International Settlements, Basel, Switzerland; and CESifo, Munich, Germany.

1 Kose et al. (2020) note that theoretical studies have focussed on investigating how public and private information is used by economic agents in formulating inflation expectations.

This paper analyses the determinants of survey-based short-term inflation expectations of professionals in the euro area, using dynamic cross-country panel estimation for 16 euro area member countries from the first quarter of 2000 to the first quarter of 2021. We use survey-based CPI inflation expectations of professionals, since they are available on a comparable basis for the countries in our sample, and since they are not distorted by risk and liquidity premia, in contrast to financial market-based measures.[2]

We find that country-specific food CPI inflation has a significant positive effect on professionals' survey-based inflation expectations in the euro area. This effect is larger than that of energy CPI inflation and of oil and global food commodity prices. We also find that depreciations of the nominal effective exchange rate lead to significantly higher inflation expectations. Moreover, inflation expectations in the euro area are persistent and the output gap has significant positive effects.

## Data

We use data on professionals' survey-based CPI short-term inflation expectations. These are taken from Consensus Economics surveys for next-year CPI inflation expectations.

Data on headline consumer price indices (CPI) comes from Datastream and national sources. Data on food CPI indices, energy CPI indices and core CPI indices are based on data from the OECD, national data and Bank for International Settlements (BIS) estimations. Core CPI inflation is defined as excluding food and energy. Brent oil prices (quarterly averages, US dollar per barrel) are from Datastream. Global commodity prices are taken as the IMF all commodity price index. Global food commodity prices are taken as the UN FAO food nominal price index. As a measure of global freight prices we use quarterly averages of the Baltic Dry Index, as reported daily by the Baltic Exchange in London, from Datastream (in US dollar per points). This index provides a benchmark for the price of moving the major raw materials by sea.

Data on output gaps (as a percentage of potential GDP) was obtained from the OECD, and is linearly interpolated from annual data. Nominal effective exchange rate indices (broad indices, quarterly average) are from the BIS, with an increase reflecting an appreciation of the domestic currency.

We consider the following 16 euro area member countries: Austria, Belgium, Estonia, Finland, France, Germany, Greece, Ireland, Italy, Latvia, Lithuania, the Netherlands, Portugal, Slovakia, Slovenia and Spain. The sample period is from 2000Q1 to 2021Q1 at quarterly frequency.

## Method and results

We study the determinants of inflation expectations by estimating the following dynamic fixed effects panel regressions, using a panel of 16 euro area member countries:

$$\pi^e_{it} = \rho\pi^e_{it\text{-}1} + \mu\Delta NEER_{it} + \gamma_f\pi^{food\ CPI}_{it} + \gamma_e\pi^{energy\ CPI}_{it} + \gamma_c\pi^{core\ CPI}_{it} + \phi\ outputgap_{it} + \chi_c\pi^{commodity}_t + \alpha_i + \varepsilon_{it}, \qquad (1)$$

where $\pi^e_{it}$ denotes next-year CPI inflation expectations from Consensus Economics surveys in percent; $\pi^{food\ CPI}_{it}$ and $\pi^{energy\ CPI}_{it}$ denote year-on-year CPI food price inflation and CPI energy price inflation, respectively, in percent, in country $i$ at time $t$; $outputgap_{it}$ denotes the output gap; $\Delta NEER_{it}$ is the quarter-on-quarter change in the nominal effective exchange rate in percent, calculated from the log change in the nominal effective exchange rate, with an increase indicating an appreciation of the domestic currency; $\pi^{commodity}_t$ are year-on-year changes in global commodity prices; finally, $\alpha_i$ are country fixed effects to control for observed and unobserved country heterogeneity. We use robust standard errors clustered at the country level.

Equation (1) is our baseline specification and the results are shown in column I of Table 1. We find that country-specific food CPI inflation has a significant positive effect on professionals' survey-based inflation expectations. A ten percentage point increase in food CPI inflation leads to an increase in inflation expectations of around 0.5 percentage points. By contrast, energy CPI inflation has no significant effect. We also find that depreciations of the domestic exchange rate in nominal effective terms have a significant positive effect on inflation expectations. A 10% depreciation over the quarter of the domestic exchange rate in nominal effective terms leads to an increase in inflation expectations of around 0.7 percentage points. Moreover, we find that the output gap has significant positive effects on inflation expectations. These effects are in addition to those of lagged inflation expectations. The inflation expectations are highly persistent, with a coefficient of around 0.7 on lagged inflation expectations.

For robustness, we also estimate versions of equation (1) where we add core CPI inflation, $\pi^{core\ CPI}_{it}$, and changes (year-on-year) in global freight prices, $\pi^{freight}_t$. These re-

2 The advantages and disadvantages of survey- and market-based measures of inflation expectations are discussed in Galati et al. (2011).

Table 1
**Short-term inflation expectations**

| Short-term inflation expectations<br>Dep. var.: $\pi^e_{it}$ | I | II | III | IV | V |
|---|---|---|---|---|---|
| $\pi^e_{it-1}$ | 0.6821*** | 0.6667*** | 0.6838*** | 0.7599*** | 0.7657*** |
| $\Delta NEER_{it}$ | -0.0678** | -0.0678** | -0.0697** | -0.0721** | -0.0507** |
| $\pi^{food\ CPI}_{it}$ | 0.0483*** | 0.0471*** | 0.0489*** | | |
| $\pi^{energy\ CPI}_{it}$ | -0.0091 | -0.0095 | -0.0080 | | |
| $\pi^{core\ CPI}_{it}$ | | 0.0207 | | | |
| *output gap*$_{it}$ | 0.0217*** | 0.0192*** | 0.0213*** | 0.0177*** | 0.0235*** |
| $\pi^{food}_{t}$ | | | | 0.0062** | |
| $\pi^{oil}_{t}$ | | | | 0.0020*** | |
| $\pi^{commodity}_{t}$ | 0.0076*** | 0.0077*** | 0.0068*** | | 0.0064*** |
| $\pi^{freight}_{t}$ | | | 0.0003 | | |
| constant | 0.5387*** | 0.5339*** | 0.5337*** | 0.4542*** | 0.4495*** |
| observations | 1082 | 1082 | 1082 | 864 | 1082 |
| number of countries | 16 | 16 | 16 | 16 | 16 |
| $R^2$ within | 0.839 | 0.839 | 0.839 | 0.795 | 0.820 |
| $R^2$ between | 0.977 | 0.979 | 0.977 | 0.989 | 0.980 |

Note: Fixed effects panel estimation; sample period: 2000Q1-2021Q1. Robust standard errors clustered at the country level. ***/**/* denote statistical significance at the 1%/5%/10% confidence level, respectively.

Source: Author's calculations.

sults are shown in columns II and III of Table 1, respectively. We find that core CPI inflation has no explanatory power over and above the output gap. Moreover, the effects of changes in global freight costs are insignificant. The effects of exchange rate depreciations, food CPI inflation and the output gap remain significant and of similar magnitude as in the baseline specification of column I.

We also estimate the following specification, where we use changes in global food commodity prices and in oil prices, instead of country-specific food and energy CPI inflation,

$$\pi^e_{it} = \rho\pi^e_{it\text{-}1} + \mu\Delta NEER_{it} + \phi outputgap_{it} + \chi_f\pi^{food}_t + \chi_o\pi^{oil}_t + \alpha_i + \varepsilon_{it}. \qquad (2)$$

Here, $\pi^{food}_t$ denotes changes (year-on-year) in global food commodity prices, and $\pi^{oil}_t$ denotes changes (year-on-year) in oil prices in percent. The results of equation (2) are shown in column IV. We find that the effect of global food prices is positive and significant, but smaller than that of country-specific food CPI inflation. Oil prices also have a small significant positive effect on inflation expectations. Again, exchange rate depreciations and the output gap have significant positive effects on inflation expectations of a very similar magnitude to those for the baseline specification in equation (1).

We also estimate a version of equation (2) where we consider changes in global commodity prices instead of oil prices and global food prices. These results are shown in column V of Table 1. We find again that global commodity prices have a significant positive effect on inflation expectations, with a similar magnitude to the baseline specification shown in column I.

## Conclusions

This paper analysed the determinants of short-term inflation expectations in the euro area based on surveys of professionals, using dynamic cross-country panel estimation for 16 euro area member countries. We find that country-specific food CPI inflation has a significant positive effect on professionals' survey-based inflation expectations. A ten percentage point increase in food CPI inflation leads to an increase in inflation expectations of around 0.5 percentage points. This effect is larger than that of global food prices. Oil prices and global commodity prices also have a significant positive effect.

We also find that depreciations of the domestic exchange rate in nominal effective terms lead to significantly higher inflation expectations. A 10% depreciation over a quarter of the domestic exchange rate in nominal effective terms leads to an increase in inflation expectations of around 0.7 percentage points. Moreover, inflation expectations in the euro area are persistent and positively affected by the output gap.

These results on the drivers of inflation expectations are relevant for monetary policy, since euro area inflation has been rising strongly in the wake of the COVID-19 pandemic. Additionally, there are concerns of possible secondround effects, with higher inflation leading to higher inflation expectations, which in turn lead to higher inflation. This could result in more persistent inflation increases. Such second-round effects would make it more difficult and costly for monetary policy to bring inflation back down to target.
